# Current pharmacological treatment of fibromyalgia- a literature review

**DOI:** 10.1192/j.eurpsy.2021.1162

**Published:** 2021-08-13

**Authors:** O. Vasiliu

**Affiliations:** Psychiatry, University Emergency Central Military Hospital Dr. Carol Davila, Bucharest, Romania

**Keywords:** fibromyalgia, gabapentinoids. serotonin and norepinephrine reuptak einhibitors, pain disorders

## Abstract

**Introduction:**

Fibromyalgia is a chronic condition, with a high degree of psychiatric comorbidity and an insufficiently explained pathogenesis. Therefore, its therapeutic management is challenging, with both pharmacological and non-pharmacological approaches being suggested as treatment options.

**Objectives:**

To analyze the current level of evidence in favour of pharmacological treatment for fibromyalgia.

**Methods:**

A literature review was performed through the main medical databases using the search paradigm “fibromyalgia” AND “pharmacological therapy” OR “antidepressants” OR “moodstabilizers” OR “anxiolytics”. All papers published between January 2000 and August 2020 were included in the primary analysis.

**Results:**

A gradually increasing interest for the treatment of fibromyalgia has been observed in the last decade, and the number of clinical trials for this indications has almost doubled in this period, when compared to the previous decade. Pregabalin, duloxetine, and milnacipran are the most supported by evidence pharmacological treatments for fibromyalgia, especially for the pain component. Amitriptyline, gabapentin, cyclobenzaprine, and tramadol have also been studied in various clinical trials, but tehre are less evidence to support their use. Cognitive dysfunctions, sleep disorders, and mood disturbances benefit from far less investigation in clinical trials, therefore no clear recommendation can be made regarding the superiority of an agent over another.

**Conclusions:**

The pain component of fibromyalgia benefits from treatment with pregabalin, duloxetine, and milnacipran, while the affective component and the cognitive dimension still need more research from the psychopharmacological perspective.

